# Biodistribution and acute toxicity of cadmium-free quantum dots with different surface functional groups in mice following intratracheal inhalation

**DOI:** 10.7150/ntno.42786

**Published:** 2020-05-18

**Authors:** Guimiao Lin, Ting Chen, Yongning Pan, Zhiwen Yang, Li Li, Ken-tye Yong, Xiaomei Wang, Jie Wang, Yajing Chen, Wenxiao Jiang, Shuting Weng, Xiaorui Huang, Jiajie Kuang, Gaixia Xu

**Affiliations:** 1 Base for International Science and Technology Cooperation: Carson Cancer Stem Cell Vaccines R&D Center, Shenzhen Key Lab of Synthetic Biology, Department of Physiology, School of Basic Medical Sciences Shenzhen University, Shenzhen 518060, China.; 2 Guangdong Key Laboratory for Biomedical Measurements and Ultrasound Imaging, Department of Biomedical Engineering, School of Medicine, Shenzhen University, Shenzhen, 518060, China.; 3 Center for Disease Control and Prevention of Ban'an district, Shenzhen 518101, China.; 4 School of Electrical and Electronic Engineering, Nanyang Technological University, 639798, Singapore

**Keywords:** InP/ZnS quantum dot, biodistribution, nanotoxicity, nanoparticles, biocompatibility

## Abstract

Indium phosphide/zinc sulfate (InP/ZnS) quantum dots (QDs) are presumed to be less hazardous than those that contain cadmium. However, the toxicological profile has not been established. The present study investigated the acute toxicity of InP/ZnS QDs with different surface modifications (COOH, NH_2_, and OH) in mice after pulmonary aerosol inhalation. InP/ZnS QDs were able to pass through the blood-gas barrier and enter the circulation, and subsequently accumulated in major organs. No obvious changes were observed in the body weight or major organ coefficients. Red blood cell counts and platelet-related indicators were in the normal range, but the proportion of white blood cells was altered. The InP/ZnS QDs caused varying degrees of changes in some serum markers, but no histopathological abnormalities related to InP/ZnS QDs treatment was observed in major organs except that hyperemia in alveolar septa was found in lung sections. These results suggested that the effects of respiratory exposure to InP/ZnS QDs on the lungs need to be fully considered in future biomedical application although the overall toxicity of quantum dots is relatively low.

## Background

Quantum dots (QDs) are typically engineered as colloidal semiconductor fluorescent nanoparticles (NPs) from II-IV (e.g., CdTe and CdSe) or III-V (e.g., InAs and InP) group elements. QDs have unique optical properties including strong photostability, brightness, and a broad excitation spectrum along with a narrow, size-tunable emission spectrum that makes them useful fluorescent probes for in vivo imaging 1 and real-time intracellular tracking of biomolecules 2. QDs are also being developed as carriers for the delivery of small interfering RNA to cancer cells in gene therapy 3 or of therapeutic agents in photodynamic therapy by bioluminescence resonance energy transfer 4, 5.

Although QDs have many potential applications, their potential toxicity remains a concern for physiological applications 6-8; additionally, their mass production and release into the environment may have negative ecological consequences 9, 10. Cadmium telluride (CdTe) QDs were found to induce apoptosis in a dose-dependent manner in two MDA-MB468 and MCF-7 breast cancer cell lines 11, while Cd selenide (CdSe) and CdSe core/zinc sulfide (ZnS) shell QDs caused singlet oxygen production and cytotoxicity 12. The toxicity of QDs also has been investigated by a variety of animal models 10, 13, 14.

Most toxicity studies of QDs have focused on cadmium-containing QDs since they are the most frequently used in biomedical applications owing to their excellent optical properties 15. However, the use of Cd-containing QDs carries the risk of toxic Cd^2+^ release 16, 17. Cd^2+^ released from CdTe QDs intravenously injected into mice caused time-dependent damage to liver and kidney 16, and another study showed that nearly 20% of Cd was released from CdS QDs within 24 h of injection, which had toxic effects 17. As an alternative, researchers have begun to develop non-Cd QDs using III-V groups of elements such as indium phosphide (InP), In arsenide (InAs), and copper In disulfide (CuInS_2_). InP/ZnS QDs are the most common core/shell QDs and are regarded as safer than those containing Cd^18^, although their toxicity remains unclear. One study investigating the toxicity profile in cell lines showed that a considerable amount of superoxide and a small amount of hydroxyl radical were generated by InP/ZnS QDs 19 and that the toxicity of InP/ZnS QDs varied across cell lines according to the efficiency of uptake, which lower overall than CdTe or CdSe/ZnS QD uptake 19. InP/ZnS QDs showed strong binding to human serum albumin, suggesting potentially toxic physiological effects 20. In fact, InP/ZnS QDs had teratogenic effects in and was lethal to developing Chinese rare minnow embryos, but did not show obvious genotoxicity 21. We previously investigated the long-term in vivo toxicity of PEGylated phospholipid-encapsulated InP/ZnS QDs in BALB/c mice and showed that they did not cause obvious toxicity in vivo following intravenous administration 22.

In vivo toxicological studies of InP/ZnS QDs have used intravenous injection as the delivery route and there is little information regarding the effects of exposure to these NPs through the respiratory route, which is possible in occupational settings. However, one study found that this resulted in persistent inflammation and granuloma formation in mouse lung 23. The biocompatibility of QDs may be improved by altering their surface functionalization; for example, it was previously reported that carboxyl (-COOH) and amino (-NH_2_) groups coated on the surface of influenced the deposition and toxicity of QDs in animal lungs.

In the present work, we investigated the biodistribution and acute toxicity of Cd-free InP/ZnS QDs with different surface functional groups (-COOH, -NH_2_, and hydroxyl [-OH]) in mice following intratracheal administration. We found that the InP/ZnS QDs—especially those modified with -OH-accumulated in the lung as well as in the other major organs while having no major toxic effects except for acute congestion of the lungs. Thus, InP/ZnS QDs with appropriate surface modification are relatively safe for biomedical applications, the effects of respiratory exposure to InP/ZnS QDs on the lungs need to be fully taken into consideration.

## Materials and methods

### Preparation and characterization of InP/ZnS QDs

InP/ZnS QDs terminated with carboxyl, amino and hydroxyl surface groups (InP-COOH, InP-NH_2_ and InP-OH) were purchased from Najingtech Company, China. These InP/ZnS QDs were originally in toluene solvent and were transferred to water by surfactant. The morphology images of InP/ZnS QDs in toluene solvent were obtained with a transmission electron microscope (TEM) (Tecnai G2 F20 S-TWIN, FEI, USA) operating at an accelerating voltage of 200 kV at room temperature. Before experiment, ICP-MS (7500C1, Agilent, USA) analysis confirmed that the indium concentrations of three QDs at same concentrations were equal. The absorption spectra of these InP/ZnS QDs were determined using a UV-Vis spectrophotometer (Cary 5000, Agilent, USA). The photoluminescence emission spectra were measured using a fluorescence spectrophotometer (F-4600, Hitachi, Japan) with an excitation wavelength of 400 nm. The hydrodynamic size distribution of these InP/ZnS QDs was obtained by a dynamic light scattering (DLS) machine (Zetasizer Nano ZS, Malvern, UK).

### Animal inhalation and weight measurements

BALB/c female mice of 6 weeks of age were purchased from Laboratory Animal Center of Guangdong Province and housed 5 per cage in a 12 h/12 h light/dark cycle. The project and animal protocols were approved by the Laboratory Animal Ethics Committee of Shenzhen University (Permit NO.201512022). The mice were anesthetized with pentobarbital sodium solution and placed on a mouse intubation platform in order to make the QDs administration faster, safer and easier. Buffered 50 μL InP/ZnS QDs (25 mg/kg body weight) or normal saline were aerosol inhaled intratracheally using a microsprayer purchased from Beijing Huironghe Technology Company. At different periods after aerosol inhalation (0 to 15 days), the mice were weighed and observed for behavioral changes.

### Frozen section and fluorescent imaging

Twenty-four hours after aerosol inhalation, mice from each group were sacrificed. The fresh major organs (heart, liver, spleen, lung, kidney) were dissected, and organ index was calculated (organ weight/body weight). Tissue sections were quickly embedded by optimal cutting temperature compound (OCT). Frozen section was prepared using a freezing microtome (CM3050S, Leica, Germany) and immediately imaged by a fluorescent microscope (BX51, Olympus, Japan).

### Inductively coupled plasma mass spectrometry (ICP-MS) analysis

At different time periods after administration, mice of each group were sacrificed. Blood samples and the lungs were collected and digested with 4 mL 65 % HNO_3_ and 1 mL 30 % H_2_O_2_ at 200 °C for 30 minutes by a microwave digestion system (Milestone ETHOS ONE, ITA). After digestion, the samples were diluted 10:1 using deionized water in order to reduce the concentration of HNO_3_. The standard of indium concentrations was prepared and the indium element content in the samples was determined by ICP-MS (7500C1, Agilent, USA) analysis.

### Blood routine examination and serum biochemistry

On Day 1 and Day 15 after aerosol inhalation, blood samples are harvested from mice treated with InP/ZnS-COOH, InP/ZnS-NH_2_, InP/ZnS-OH s and mice treated with saline buffer. Blood routine examination of whole blood was performed using the whole blood by a routine blood test instrument (RJ-0C107223, Mindray, China). Blood serum was separated and serum biochemistry was analyzed by blood biochemistry analyzer (Mindray BS-220).

### Hematoxylin and eosin staining

Before the sacrifice, the mice were given heart perfusion to remove the blood in the vessels. For histological analysis, the lungs were harvested from mice and fixed with 10% neutral buffered formalin for 48 hours. Cut into small blocks, the lung tissues were dehydrated with alcohol solutions and hyalinized with xylene solution. After hyalinization, the lung tissues were embedded in paraffin, sectioned, and stained with hematoxylin and eosin (H & E). The H & E staining slices were examined under a light microscope (BX51, Olympus, Japan) by a clinical pathologist.

### Statistical analysis

All experimental data were presented as mean ± standard deviation (SD). Multigroup comparisons of the means were performed by one-way analysis of variance (ANOVA) test. Dunnett's test was used to analyze the differences between the experimental groups and the control group. All statistical calculations were carried out using SPSS 11.0 software package. P < 0.05 was considered to indicate significant difference statistically.

## Results

### Characterization of InP/ZnS QDs

**Figure 1:**
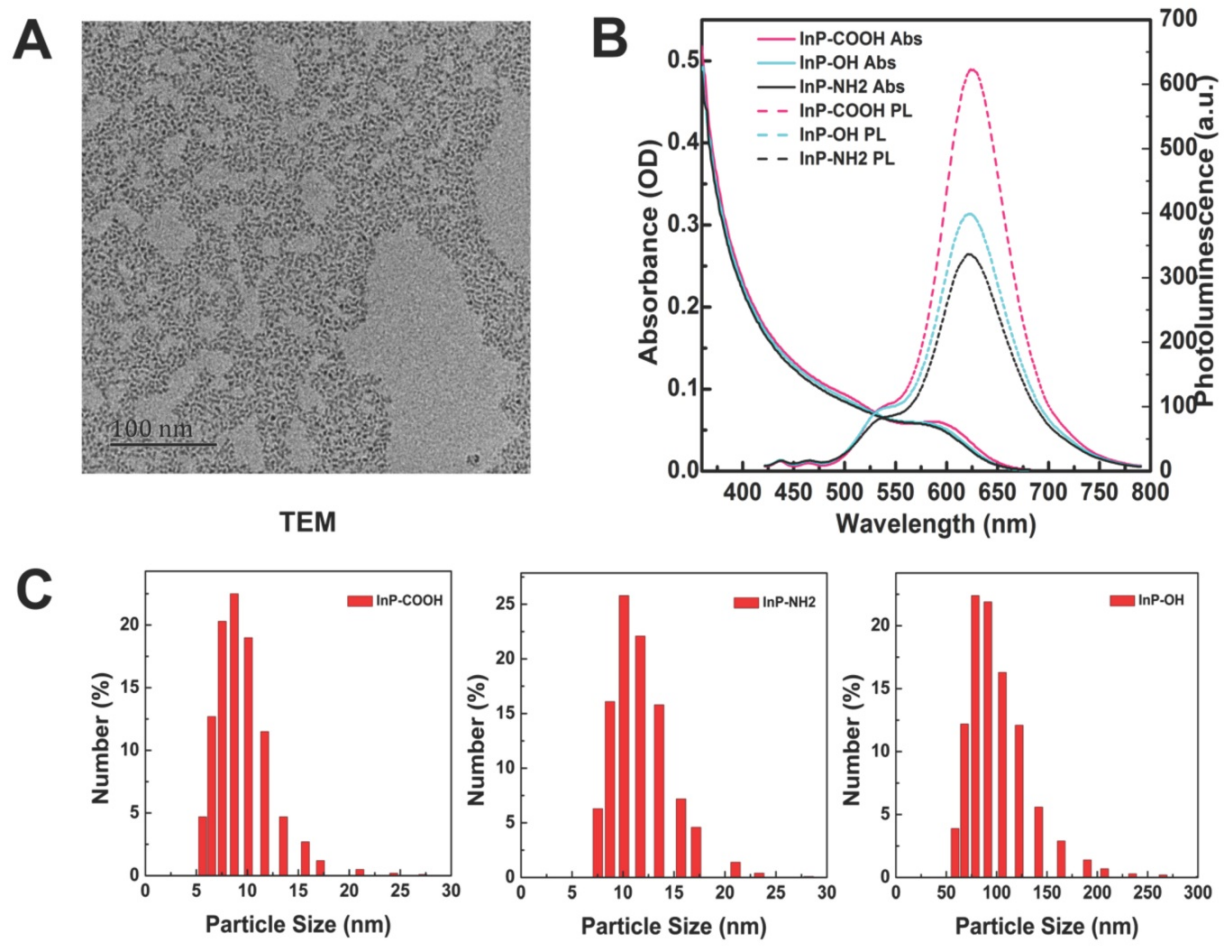
Characterization of InP/ZnS QDs with different surface functional groups. (A) Representative transmission electron micrograph of InP/ZnS QDs dispersed in toluene. Scale bar = 50 nm. (B) Absorption and photoluminescence (PL) spectra of InP/ZnS-COOH, InP/ZnS-NH_2_, and InP/ZnS-OH QDs. (C) Hydrodynamic size distributions of InP/ZnS-COOH, InP/ZnS-NH_2_, and InP/ZnS-OH QDs dispersed in deionized water.

The morphology of InP/ZnS QDs before surface modification was examined by transmission electron micrograph (Fig. 1A). The InP/ZnS QDs had an average size of 5-6 nm and were moderately monodispersed. After surface modification, the hydrodynamic diameter and zeta potential of aqueous InP/ZnS QDs were determined by DLS. The hydrodynamic diameters of InP/ZnS-COOH, InP/ZnS-NH_2_, and InP/ZnS-OH QDs were 10.29 ± 3.74, 13.62 ± 3.53, and 104.00 ± 32.74 nm, respectively; and the zeta potentials were -46.00 ± 6.72, -43.8 ± 7.17, and -54.00 ± 5.49 mV, respectively (Fig. 1C). The InP/ZnS QDs showed similar absorption spectra with an absorption peak at around 580 nm; and the photoluminescence spectra were symmetrical, with an emission peak at 625 nm after excitation by a 400-nm light source (Fig. 1B).

### Effect of InP/ZnS QDs on body weight and major organ coefficients

**Figure 2:**
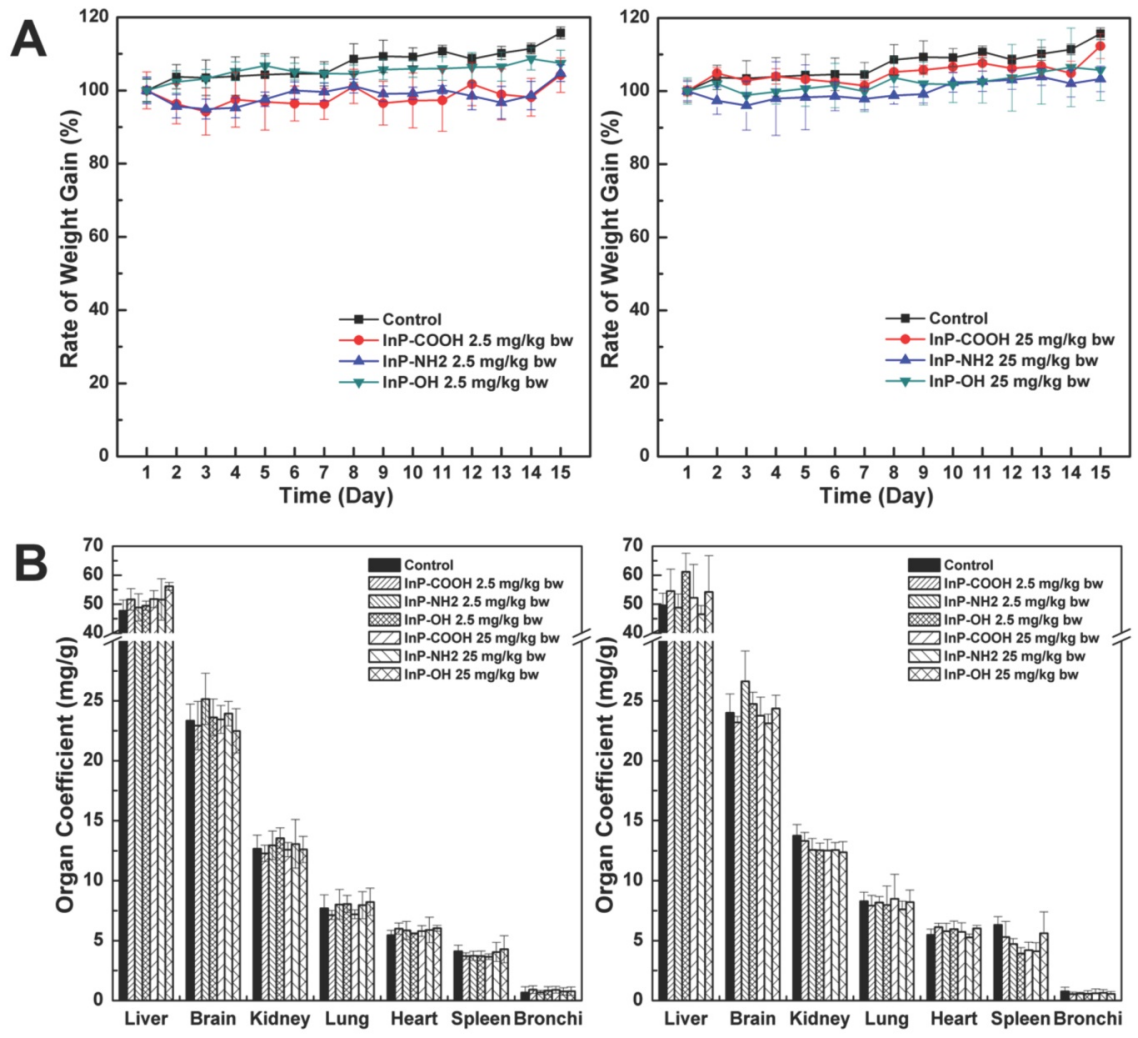
Body weight and major organ coefficients of mice after pulmonary aerosol inhalation of InP/ZnS QDs. (A) Body weight after inhalation of InP/ZnS QDs at doses of 2. and 25mg/kg; (B) Major organ coefficients were calculated 24 h and 15 days after exposure to InP/ZnS QDs (n = 6).

The body weight of mice was continually monitored to assess the toxicity of InP/ZnS QDs. There were no differences in body weight between treatment and control groups at 15 days after InP/ZnS QD administration (P < 0.05; Fig. 2A). The mice were sacrificed 15 days after QD inhalation and the major organ coefficients were determined. There were no differences in the coefficients between the treatment and control groups (P < 0.05; Fig. 2B). In addition, during this 15-day period there were no changes in behavior (drinking/eating) or physical appearance (e.g., hair color and glossiness) in mice treated with QDs. These results suggest that InP/ZnS QDs are biocompatible and well tolerated by mice.

### In vivo distribution of QDs in major organs and clearance

**Figure 3:**
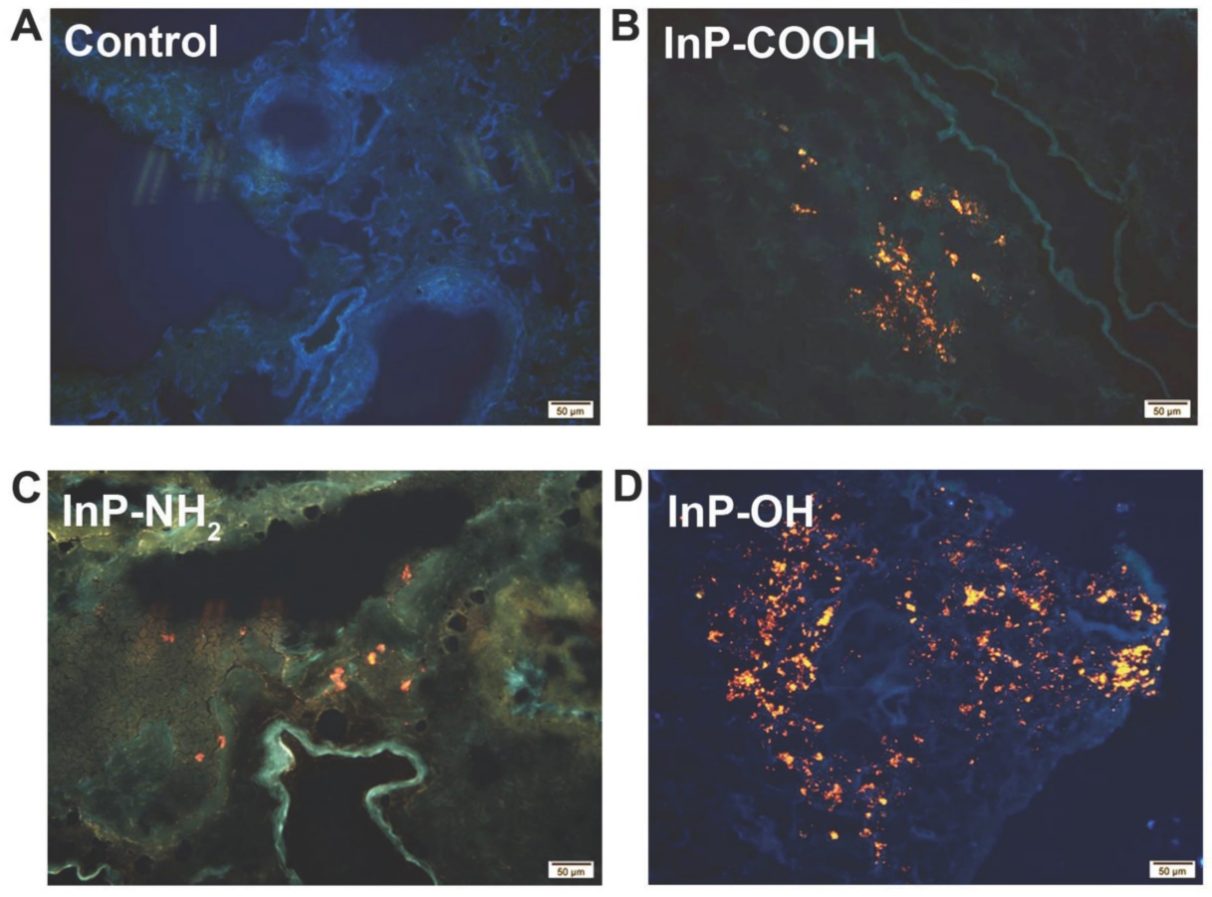
Fluorescence micrographs of lung tissue sections from mice 24 h after intratracheal inhalation of InP/ZnS QDs. (A) Control; (B) InP-COOH; (C) InP-NH_2_; (D) InP-OH.

The biodistribution of QDs can provide important information on the physiological behavior of QDs such as site of accumulation and clearance route. In order to investigate the biodistribution of the QDs, lung tissue was harvested for fluorescence imaging 24 h after administration of 25 mg/kg QDs. The strong fluorescence of the QDs was detected in the lung tissue of QD-treated but not control mice (Fig. 3). These results indicate that the QDs can label organs without quenching of the fluorescence signal.

To quantify the QDs accumulated in the respiratory system, we measured In concentration in lung and trachea by ICP-MS. At 2 h after aerosol inhalation, In concentrations in mice treated with InP-COOH, InP-NH_2_, and InP-OH were 23387.2943 ± 4785.9052, 37122.8606 ± 5920.6531, and 53032.5783 ± 11286.5269 ng/g, respectively; after 24 h, the concentrations had decreased to 4235.8155 ± 1314.5845, 920.6531 ± 118.1000, and 2565.9595 ± 565.7735 ng/g, respectively. This suggests that most of the QDs are cleared from the lung within 24 h. It worth noting that lung In concentration was higher in mice treated with InP-OH QDs than in those treated with the other two QDs, suggesting that InP-OH QDs has a lower capacity to penetrate the blood-gas barrier.

**Figure 4:**
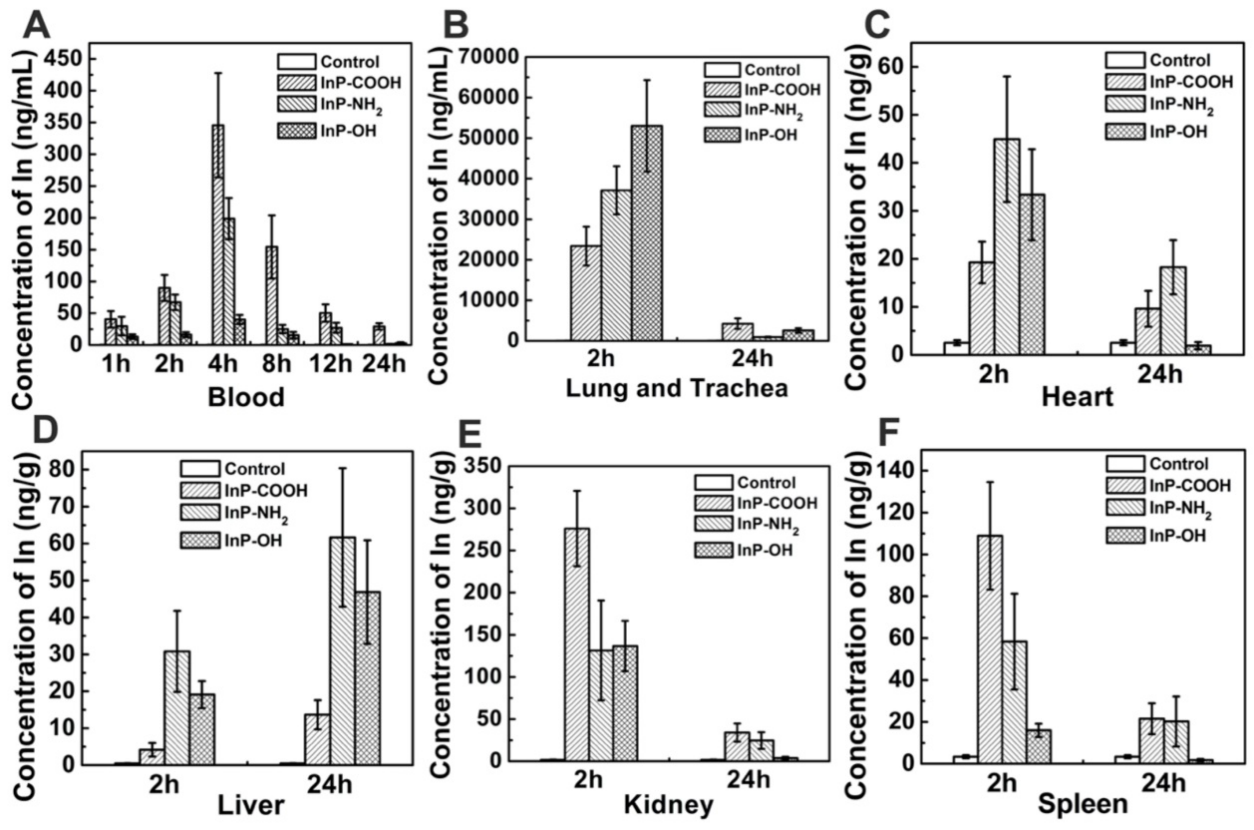
Accumulation of In in blood and other major organs of mice exposed to InP/ZnS QDs (n = 4). (A) Blood. (B) Lung and trachea. (C) Heart. (D) Liver. (E) Kidney. (F) Spleen.

A major clearance route of substances from the lung is passage through the blood-air interface and diffusion into the circulation. In order to investigate the clearance route of the InP QDs, In concentrations in the blood were analyzed by ICP-MS at 1, 2, 4, 8, 12, and 24 h post administration. In from QDs accumulated in the blood in the first 4 h and was detected for up to 24 h, but the concentration gradually decreased thereafter (Fig. 4A). These results suggest that QDs cross the blood-air interface, enter the circulation, and are cleared from the body. We also examined whether QDs accumulate at other sites in the body and found that at 2 h post administration, In was present in all major organs including heart, liver, kidney, spleen, and brain, with the highest concentrations in kidney for all three types of QD at the 2-h time point. The In concentration in kidney of mice treated with InP-COOH QDs (275.9907 ± 44.7784 ng/g) was higher than those of mice treated with InP-NH_2_ QDs (131.4337 ± 59.2375 ng/g) and InP-OH QDs (136.7318 ± 29.8466 ng/g). The spleen also accumulated a large amount of In after 2 h, with concentrations of 108.9116 ± 25.6811, 58.3739 ± 22.8358, and 15.9515 ± 3.1718 ng/g for InP-COOH, InP-NH_2_, and InP-OH QDs, respectively. After 24 h, In concentration decreased in kidney, heart, spleen, and brain but increased in liver from 4.1834 ± 1.8327 to 13.6443 ± 3.9456 ng/g for InP-COOH QDs, from 30.7993 ± 10.9765 to 61.6554 ± 18.7495 ng/g for InP-NH_2_ QDs, and from 19.1280 ± 3.6708 to 46.861 ± 14.0314 ng/g for InP-OH QDs.

### Routine blood test

**Figure 5:**
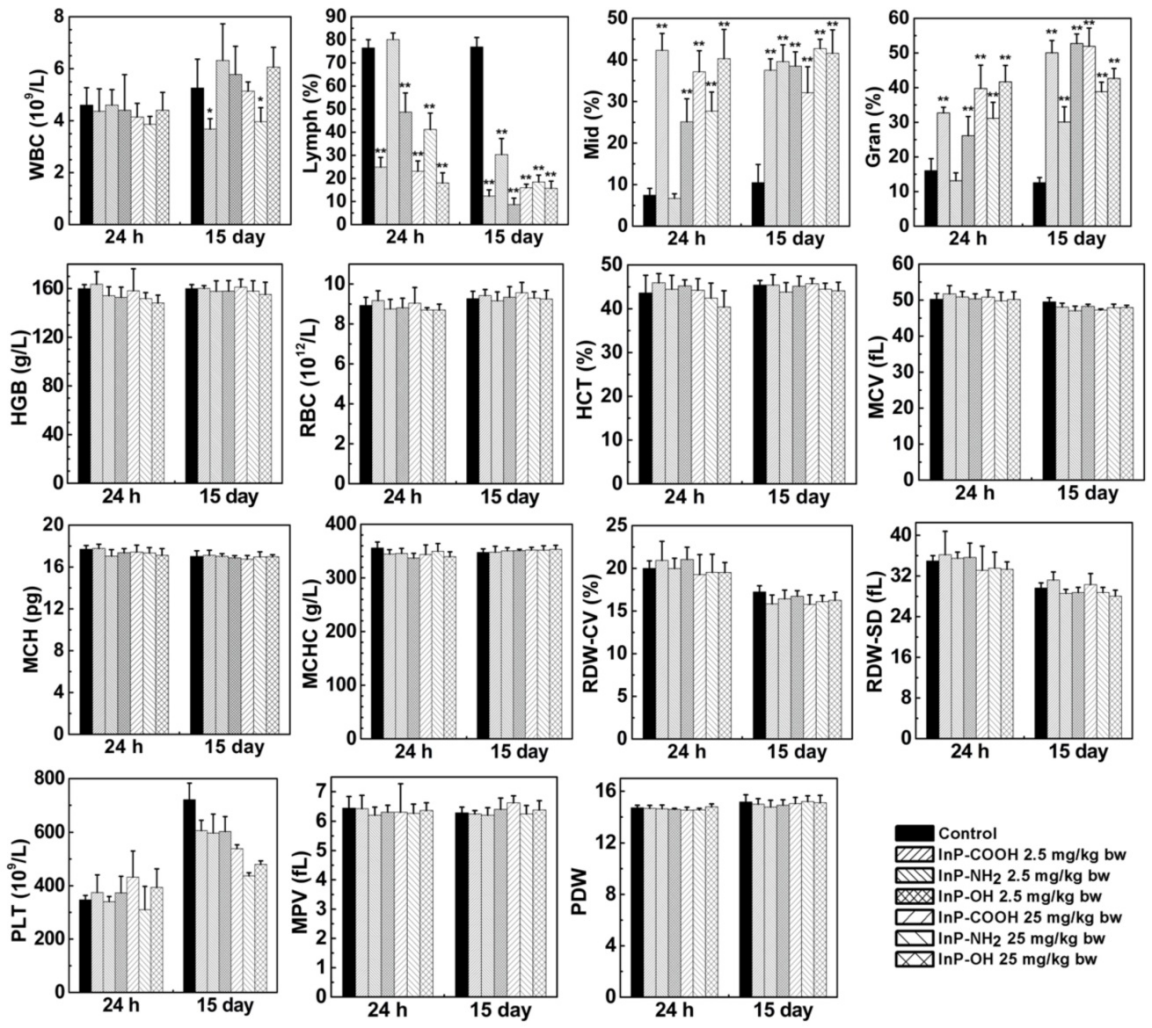
Expression of haematological markers 24 h or 15 days after InP/ZnS QD treatment (n = 6). *P < 0.05, **P < 0.01. Gran, granulocyte; HCT, hematocrit; HGB, hemoglobin; Lymph, lymphocyte; MCH, mean corpuscular hemoglobin; MCHC, mean corpuscular hemoglobin; MCV, mean corpuscular volume; Mid, middle cell; MPV, mean platelet volume; PDW, platelet distribution width; PLT, platelet; RBC, red blood cell; RDW-CV, coefficient of variation of RBC volume distribution width; RDW-SD, standard deviation of RBC volume distribution width; WBC, white blood cell.

When QDs enter the bloodstream, they interact with blood cells and may alter blood biochemical composition. We carried out blood routine examination of mice treated with InP QDs or normal saline by measuring blood biomarkers including white blood cell count; lymphocyte percentage; middle cell percentage; granulocyte percentage; hemoglobin; red blood cell count; hematocrit; mean corpuscular volume; mean corpuscular hemoglobin; mean corpuscular hemoglobin concentration; coefficient of variation of RBC volume distribution width; standard deviation of RBC volume distribution width; platelet; mean platelet volume; and platelet distribution width at 24 h and 15 days after treatment. We found that all measured parameters were within normal ranges except that QD treatment decreased lymphocyte count while increasing granulocyte and middle cell counts (Fig. 5). This suggests that inhaled InP/ZnS QDs can elicit a systemic immune response in mice.

### Serum biochemical parameters

After clearance from the circulation, InP/ZnS QDs move to tissues and organs. Exogenous substances are metabolized in the liver and cleared through the kidney. To investigate the effects of InP/ZnS QD accumulation in liver and kidney, we measured serum levels of biomarkers related to the function of these two organs including total protein, albumin, globulin, albumin to globulin ratio, alanine transaminase, aspartate transaminase, alkaline phosphatase, uric acid, urea, creatinine, triglyceride, total cholesterol, creatine kinase, lactate dehydrogenase, and α-hydroxybutyrate dehydrogenase. As shown in Figure 5, the InP/ZnS QDs caused varying degrees of changes in some serum markers. On Day 15, there is no significant difference in UREA, CK, CREA, and UA between control group and InP/ZnS QDs-treated group. However, InP/ZnS QDs treatment significantly reduced the levels of TP, ALB, and ALP. In addition, the levels of GLB and GLB also remarkably decreased in high-dose treated group. These results suggest that InP/ZnS QDs accumulation interferes with the function of the organs to some extent.

### Histopathological analysis

**Figure 7:**
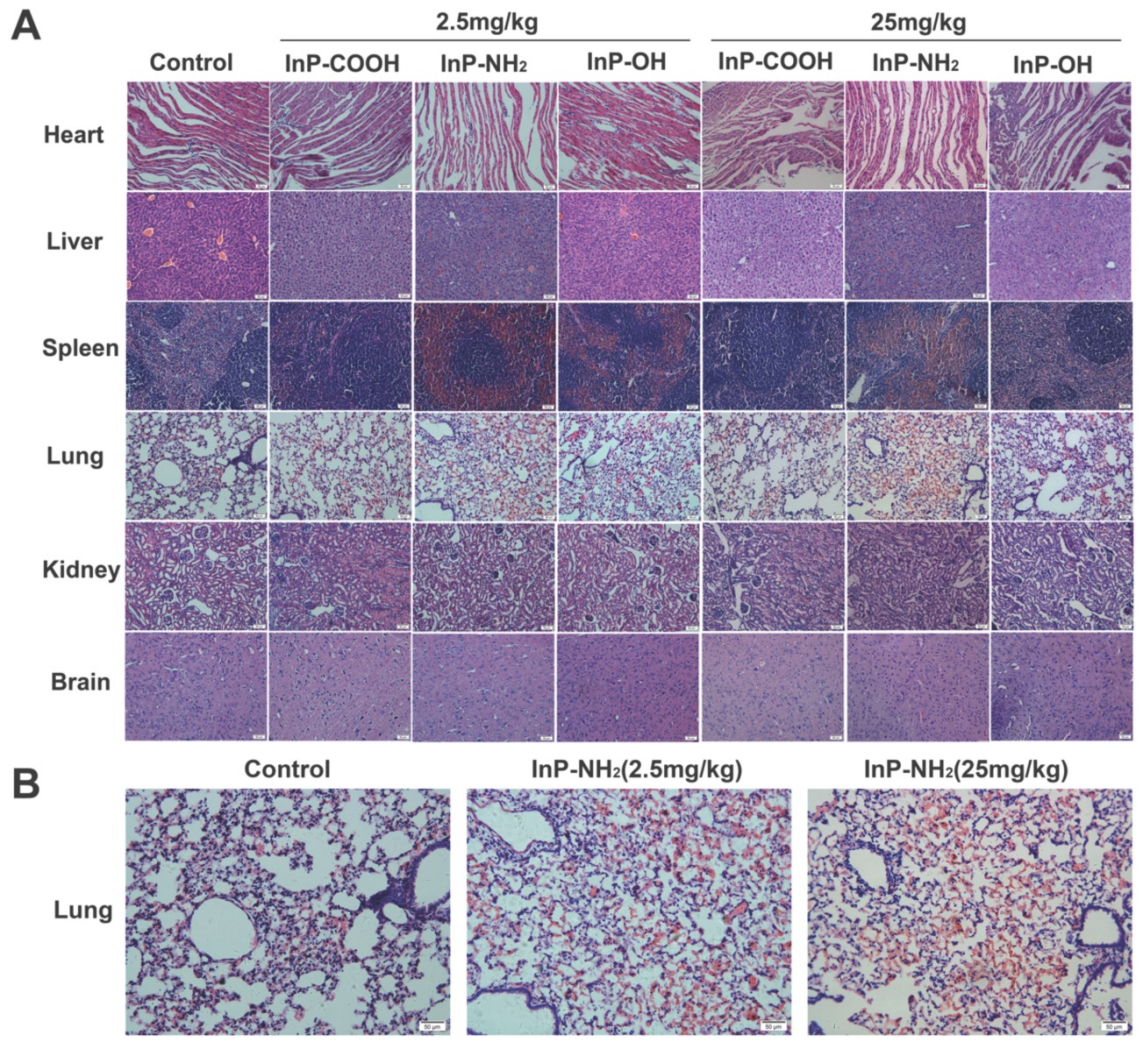
H&E staining of lung, kidney, liver, spleen, heart, and brain tissue sections. Scale bar, 50 μm. (A) Representative micrographs of H&E staining of major organs. (B) Enlarged view of lung tissue sections from control and InP-NH_2_ QD-treated mice.

To investigate whether QD accumulation in the various organs causes histological changes, lung, kidney, liver, spleen, heart, and brain tissue sections were examined by hematoxylin and eosin (H&E) staining 15 days after treatment with QDs at concentrations of 2.5 and 25 mg/kg. There were no histopathological abnormalities observed in any of the organs of QD-treated mice (Fig. 7A); however, significant hyperemia in alveolar septa was observed in the lung of mice treated with InP-NH_2_ QDs, although there were no other pathological changes such as fibrosis or necrosis (Fig. 7B). These results indicate that InP/ZnS QDs that cross the gas-blood barrier are relatively non-toxic to the major organs.

## Discussion

With the quick reparation and widespread use of engineered NPs in every field 24, 25, the risks associated with exposure to NPs are becoming an increasing concern for human health 26-29. In particular, NP dust in the environment as well as in laboratories and factories can enter the body through the respiratory tract 30, potentially causing lung injury and other disorders 31, 32. For instance, short-term inhalation of copper oxide NPs (CuO) resulted in the upregulation of chemokine C-C motif chemokine ligand 2, the oncoprotein epithelial cell transforming 2, and proinflammatory factors in the bronchoalveolar epithelium of rats 33. Zinc oxide, nickel oxide, and ceric dioxide NPs caused lung injury in rats by inducing free radical generation in lung tissue following inhalation and intratracheal instillation 34.

In this study, the acute toxicity of InP/ZnS QDs with different surface modifications (COOH, NH_2_ and OH) in mice after intratracheally inhalation at a single dose of 2.5mg/kg or 25 mg/kg was investigated. The different surface functional group of QDs makes them different in water stability and hydrodynamic size distributions. The InP/ZnS-OH QDs we used here were much easier to aggregate than the other two InP/ZnS QDs coated with carboxyl or amino group, and the particle size of InP/ZnS-OH QDs in the water was far greater than the others.

Our data demonstrated that all these three InP/ZnS QDs were able to enter in the lung. It is well known that the deposition of inhaled particles in the respiratory tract depends on their size. Particles between 2.5 and 10 µm in size normally deposit in upper respiratory tract. Those with 0.1-2.5 µm are prone to deposit in lower respiratory tract. Particles less than 0.1 µm were easily deposited in distant respiratory tract, such as the terminal bronchioles and alveoli 30. Although all these QDs accumulated in the lung, the amount of QDs differed in lung deposition, with InP/ZnS-OH being the most abundant which was possibly caused by their different sizes in aqueous solutions. Since the biodistribution and clearance of NPs were size-dependent, the size variety InP/ZnS QDs led to the different remove from the lungs through the gas-blood barrier. Here, the hydrodynamic diameters of InP/ZnS-COOH and InP/ZnS-NH_2_ QDs were around 10 nm, while InP/ZnS-OH QDs were prone to aggregate in the water solution that made the average hydrodynamic diameters of InP/ZnS-OH QDs around 104 nm, which was obviously bigger than the other two QDs. It was much easier for the smaller QDs to cross the gas-blood barrier and translocate to systemic circulation.

After the QDs entered the blood fluid, the QDs were found in tissues and organs, which have been reported by several researchers previously 22, 35, 36. Tt is well known that the different in vivo biodistribution profile was determined by several factors, such as size, surface chemistry and animal strains 37. For example, Su et al found that the QDs were initially absorbed in liver at 0.5-4 h post-injection, and then accumulated in the kidney during the 15-80 days observation period. Moreover, they showed that the biodistribution of QDs was size-dependent. The QDs with larger sizes were more quickly to accumulate in the spleen; on the contrary, the smaller QDs were more easily to be absorbed by kidney 38. In addition, David et al exposed male mice from 8 genetically diverse inbred strains to CdSe/ZnS QDs with an amphiphilic polymer via oropharyngeal aspiration. They revealed that the levels of Cd in lungs from some strains including CAST, AJ, 129, NZO, NOD had obviously higher levels of Cd than other strains such as B6, PWK, and WSB. CdSe/ZnS QDs showed higher lung Cd levels at 8 h and slower clearance with a longer half-life in AJ mice than B6 mice 39. Here, our data showed that the InP/ZnS QDs mainly accumulated in the kidney and spleen of BALB/c mice in the beginning and then migrate to the liver.

Once QDs entered the blood and tissue organs, they might affect the normal physiological functions of blood components and tissues. Our data suggested that QDs treatment did not indicate any toxic effects on red blood cells, but cause obvious changes to white blood cells. White blood cells are the body's defense cells, which participate in the detection and defense against bacteria, viruses and other foreign substances. The initial resistance of humans against foreign substances comes from white blood cells, so the initial change in the body's response is to increase or decrease white blood cells. The changes of white blood cells in mice treated with InP QDs suggested an inflammatory response or potential immunotoxicity. Many studies have shown that respiratory exposure to NPs caused inflammation in the body, leading to changes in white blood cells. For example, Carvalho et al revealed that instillation of multi-walled carbon nanotubes (MWCNT) into the airways of mice aroused an inflammatory response in the lung with an increase of eosinophils 40. Chien et al demonstrated that inhalation of zinc oxide NPs at occupationally relevant levels for 2 weeks caused sustained renal interstitial and periglomerular inflammation in Sprague Dawley rats 41.

In this experiment, there was no significant difference in the organs' coefficients between control and InP/ZnS QDs treated groups although these QDs were able to migrate to these organs. And the biomarkers for the functions of liver and kidney also suggested that the accumulations of these QDs in the major organs were low toxic. Consistently, previous studies on the toxicity of different QDs have found little impact of QDs on liver and kidney functions, although liver and kidney were two of the most abundant organs for QDs accumulation. For instance, researchers have showed that all measured liver and kidney factors in serum from QDs-treated Kunming mice or rhesus macaques were within normal ranges and did not suggest any toxic responses associated with phospholipid micelle encapsulated CdSe/CdS/ZnS QDs 35, 42.

The present study goes further to investigate the effect of InP/ZnS QDs on histomorphology of the major organs using H&E staining. It is well known that pathological diagnosis is the gold standard for judging organ injury. No apparent histopathological abnormalities in kidney, liver, spleen, heart and brain sections in mice were observed after treatment with InP/ZnS QDs, suggesting low toxicity of QDs to these organs. However, obvious hyperemia in alveoli septum was found in InP/ZnS-NH_2_ QDs-treated mice, indicating lung injury occurred after InP-NH_2_ QDs exposure. Alveolar septum is a thin layer of connective tissue between adjacent alveoli. In the alveolar septum, there is a dense network of continuous capillaries attached to the alveolar wall. There are more elastic fibers in the alveolar septum, and their elastic retraction can promote the retraction of the expanded alveolar. Alveolar septal hyperemia refers to the excessive filling of blood in pulmonary capillaries, which can lead to degeneration of elastic fibers. The alveolar elasticity is weakened and retraction ability is poor. Over time, alveolar enlargement will occur and pulmonary ventilation will be impaired, thus affecting the respiratory function of the lungs. In other words, InP-NH_2_ QDs, although only slight pathological changes occur after 15 days of treatment, if further progress is made, it may seriously affect the physiological function of the lungs.

It is worth mentioning that some previous studies have showed that QDs exposure did not cause pathological damage to lung tissue 22, 35, 42. The causes of lung pathologic injury depend on many factors, and one of the most important of which is the exposure route of QDs. In general, QDs were injected into the body via an intravenous route, and usually did not cause pathological changes in the lungs^22^, ^35^, ^42^. In contrast, QDs entering the respiratory tract were more likely to cause lung damage^43^. The reason is that when QDs enter the body through the respiratory tract, the lung is the earliest and most direct tissue organ that reacts with QDs. A large amount of QDs accumulates in a short period of time, which is easy to directly stimulate the lungs to suffer from oxidative stress damage and induce the lungs to experience inflammatory and other immune reactions. In addition to exposure route, our data suggested that surface modification of QDs was also an important factor affecting lung injury. In this study, only the InP/ZnS QDs with surface groups of NH_2_ showed significant alveolar septal hyperemia, while those with surface groups of OH and COOH did not show significant hyperemia. Because surface modification is of great importance to the toxicity of InP/ZnS QDs, the optimal surface modification should be considered and selected to reduce the potential adverse toxicity of InP/ZnS QDs in biological applications.

## Figures and Tables

**Figure 6:**
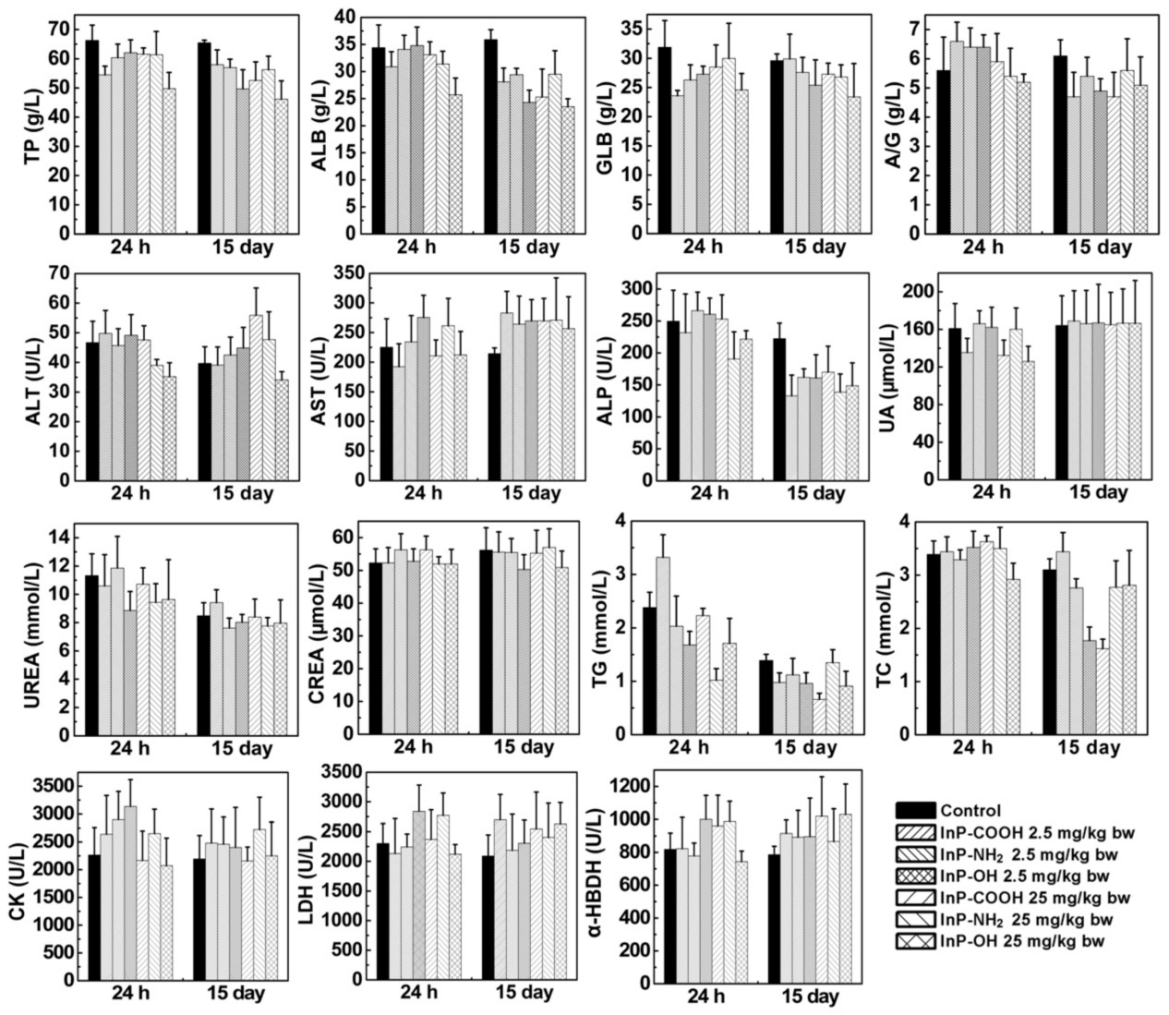
Serum biochemical biomarkers measured at indicated time points after InP/ZnS QD treatment (n = 6). *P < 0.05, **P < 0.01, ***P < 0.001. α-HBDH, α-hydroxybutyrate dehydrogenase; A/G, ratio of albumin to globulin; ALB, albumin; ALP, alkaline phosphatase; ALT, alanine transaminase; AST, aspartate transaminase; CK, creatine kinase; CREA, creatinine; GLB, globulin; LDH, lactate dehydrogenase; TC, total cholesterol; TG, triglyceride; TP, total protein; UA, uric acid.
